# Effect of Paternal Age on Reproductive Outcomes of Intracytoplasmic Sperm Injection

**DOI:** 10.1371/journal.pone.0149867

**Published:** 2016-02-22

**Authors:** Yixuan Wu, Xiangjin Kang, Haiyan Zheng, Haiying Liu, Qing Huang, Jianqiao Liu

**Affiliations:** 1 Department of Reproductive Medicine, the Third Affiliated Hospital of Guangzhou Medical University, Guangzhou, Guangdong, China; 2 Key Laboratory of Reproductive Medicine of Guangdong Province, Guangzhou, Guangdong, China; 3 Key Laboratory for Major Obstetric Diseases of Guangdong Province, Guangzhou, Guangdong, China, Key Laboratory of Reproduction and Genetics of Guangdong Higher Education Institutes, Guangzhou, Guangdong, China; Clermont-Ferrand Univ., FRANCE

## Abstract

The impact of paternal age on reproduction, especially using assisted reproductive technologies, has not been well studied to date. To investigate the effect of paternal age on reproductive outcomes, here we performed a retrospective analysis of 2,627 intracytoplasmic sperm injection (ICSI) cycles performed at the Reproductive Medicine Center of the Third Affiliated Hospital of Guangzhou Medical University (China) between January 2007 and May 2015. Effect of paternal age on embryo quality [number of fertilized oocytes, 2 pronucleus zygotes (2PNs), viable embryos, and high-quality embryos] was analyzed by multiple linear regression. Relationships between paternal age and pregnancy outcomes were analyzed by binary logistic regression. After adjusting for female age, no association between paternal age and the following parameters of embryo quality was observed: number of fertilized oocytes (B = -0.032; 95% CI -0.069–0.005; *P* = 0.088), number of 2PNs (B = -0.005; 95% CI -0.044–0.034; *P* = 0.806), and number of viable embryos (B = -0.025; 95% CI -0.052–0.001; *P* = 0.062). However, paternal age negatively influenced the number of high-quality embryos (B = -0.020; 95% CI -0.040–0.000; P = 0.045). Moreover, paternal age had no effect on pregnancy outcomes (OR for a 5-year interval), including the rates of clinical pregnancy (OR 0.919; 95% CI 0.839–1.006; *P* = 0.067), ongoing pregnancy (OR 0.914; 95% CI 0.833–1.003; *P* = 0.058), early pregnancy loss (OR 1.019; 95% CI 0.823–1.263; *P* = 0.861), live births (OR 0.916; 95% CI 0.833–1.007; *P* = 0.070), and preterm births (OR 1.061; 95% CI 0.898–1.254; *P* = 0.485). Therefore, increased paternal age negatively influences the number of high-quality embryos, but has no effect on pregnancy outcomes in couples undergoing ICSI cycles. However, more studies including men aged over 60 years with a longer-term follow-up are needed.

## Introduction

While the adverse effect of maternal age on reproductive outcomes is well studied, the effect of male aging on reproduction remains more controversial. Male aging can directly damage sperm DNA [1], increase sperm DNA methylation [2], cause sperm damage through the production of excessive reactive oxygen species [3], and compromise spermatogenesis [4]. As a result, paternal age may affect assisted reproduction technology (ART) outcomes. Although some studies have investigated the effect of paternal aging on ART outcomes, they demonstrate discrepant results. Some studies have shown that paternal age is not associated with pregnancy outcomes using conventional in vitro fertilization (IVF) techniques [5, 6], while others report decreased pregnancy and live birth rates with increased male age [7, 8]. Moreover, our previous study showed that paternal age had no impact on the fertilization rate, embryo quality at the cleavage stage, and rates of early pregnancy loss in conventional IVF, but it did have a negative effect on pregnancy and implantation rates when the maternal age was 30–34 y [9].

Intracytoplasmic sperm injection (ICSI), as an alternative to IVF, is a more invasive manipulation and limits the natural selection of the sperm. It was developed mainly to treat severe male infertility, although it is now widely used in some reproductive centers to avoid complete fertilization failure. Therefore, the effect of paternal age on reproductive outcomes in ICSI cycles may be different from conventional IVF. Indeed, in a retrospective study of 227 couples with male partners aged ≥50 y who underwent ICSI, advanced paternal age had a detrimental effect on fertilization rate, but had no impact on pregnancy rate or embryo quality at the cleavage stage [10]. Meanwhile, Ferreira et al. documented that paternal age only had an adverse effect on implantation and pregnancy rates in men with oligozoospermia [11]. Another report found no association between paternal age and pregnancy outcomes in 184 ICSI cycles using spermatozoa obtained through testicular sperm extraction [12]. Finally, a recent retrospective cohort study investigating nearly 5,000 oocyte-donation cycles reported that paternal age had no effect on pregnancy outcomes, even when adjusted for recipients’ age [13]. Based on the limited number of studies, there remains no consensus on the effect of paternal age on reproduction when investigating ICSI cycles. Therefore, to further investigate the potential effect of paternal age on reproductive outcomes using ICSI, here we retrospectively analyzed over 2,500 ISCI cycles.

## Materials and Methods

### Study population

This study is a retrospective analysis including ICSI cycles in the Reproductive Medicine Center of the Third Affiliated Hospital of Guangzhou Medical University (China) between January 2007 and October 2013. The inclusion criteria were: (1) ICSI cycles with embryo transfer between January 2007 and May 2015; (2) maternal age <39 years; (3) first cycle performed in our center; and (4) agonist or antagonist protocols used. The study was approved by the Ethics Committee of the Third Affiliated Hospital of Guangzhou Medical University. Since this is a retrospective study, patients were not asked to participate in this study.

### Semen preparation

Three types of sperm origins were included in this study: ejaculated sperm, sperm from percutaneous epididymal sperm aspiration (PESA), and sperm from testicular sperm extraction (TESA). All male patients were required to undergo 3–7 days of abstinence before providing the semen samples for fertilization. After liquefaction of the semen (about 30 min at room temperature), the sperm samples were analyzed for volume, concentration, and motility according to World Health Organization criteria (2010) [14].

Combinations of gradient centrifugation and swim-up were used for the preparation of ejaculated sperm. Semen samples were processed using a density gradient (PureSperm®, Nidacon International AB, Sweden) and sperm wash medium (Sperm Wash, Nidacon, International AB, Sweden). PureSperm gradients of 90% and 45% were used for the experiment. All procedures were conducted under sterile conditions, and all media was warmed to 37°C. Using a sterile pipette, 1.5 mL of the lower layer (90% PureSperm gradient) was transferred into a conical centrifuge tube. Using a new sterile pipette, 1.5 mL of the upper layer (45% PureSperm gradient) was gently dispensed on top of the lower layer. A liquefied semen sample was then placed on top of the upper layer and the tube was centrifuged for 20 min at 400×g. The upper and lower layers were carefully aspirated without disturbing the pellet. Using a transfer pipette, 5 mL of sperm wash medium was added and the re-suspended pellet was centrifuged for 10 min at 400×g. The supernatant was then removed and 0.3–0.5 mL of sperm wash medium was gently dispensed on the top of the pellet. The tube was then incubated at an angle of 45° for 30–60 min in the incubator at 37°C and 5% CO_2_.

To obtain the sperm by PESA, the patient was laid supine on the operating table, and the genital region was shaved with a straight razor to allow for better access and visualization of the epididymis. The area was sterilized with iodine solution, and sterile drapes were placed overtop. A local cord block anesthetic of 15 cc 2% lidocaine was injected and allowed to settle for 5 min. The epididymis was then grasped between the thumb and forefinger. A 21-gauge needle attached to a 3 cc syringe was then inserted into the epididymis. Suction was applied following insertion, and multiple passes were made without removing the needle from the initial site of insertion. The spermatozoa obtained were then placed into a sperm buffer and transported to an IVF laboratory. Epididymal spermatozoa were obtained at the same time as oocyte retrieval and used immediately for ICSI [15].

TESA was used for retrieving testicular sperm. To perform TESA, the area around the spermatic cord was locally anesthetized by injecting 5 mL of 2% lidocaine. The aspiration was then performed in the center as well as in the upper and lower poles of each testis using a 23-gauge needle with a 20 cc syringe attached to it. A constant negative pressure was applied to the syringe when the needle reached the center of the testis, and aspiration was done with gentle back and forth movements of the needle at different angles in each puncture location. TESA was subsequently performed on both testes. Tunica albugina and epididymis were exposed through a 5- to 15-mm incision in the scrotal skin and tunica vaginalis. Three different incisions were made in tunica albugina near the sites of needle insertion, and a sample of roughly 5×3×2 mm was excised from each site. The specimens were placed in separate tubes containing IVF medium, and the tubes were centrifuged for 10 min at 400×g. The upper layers were carefully aspirated without disturbing the pellets, and 0.3 mL of IVF medium was added to resuspend the pellets. The tubes were incubated at 37°C and 5% CO_2_ and until used for ICSI.

### Ovarian stimulation, ICSI, and embryo transfer

For the agonist protocol, 1.0–1.3 mg of gonadotropin-releasing hormone (GnRH) agonist (Triptorelin, Ipsen Pharma Biotech, France) was administered in the luteal phase of the previous cycle. Tests of serum estradiol (E_2_), luteinizing hormone (LH), follicle-stimulating hormone (FSH), and ultrasound were performed after 14 days of downregulation. Ovarian stimulation was started once the downregulation was satisfactory, i.e. serum E_2_ was <50 pg/mL, follicle diameter was 4–6 mm, and endometrium thickness was <5 mm. For the antagonist protocol, ovarian stimulation was started with FSH if tests of serum basal E_2,_ LH, FSH, and ultrasound indicated an early follicular stage. A GnRH antagonist (Cetrotide, Baxter Oncology GmbH, Germany) was administered once the dominant follicle was >14 mm and continued to the day of human chorionic gonadotropin (HCG) administration.

When at least 3 follicles were ≥17 mm, ovulation was triggered with HCG. Oocytes were retrieved 34–36 h later, and then incubated in culture medium covered with mineral oil for 3 h at 37°C and 6% CO_2_. Cumulus cells were removed by a 30 s exposure to HEPES-buffered medium containing 80 IU/mL hyaluronidase. Denuded oocytes were then assessed for nuclear status. Oocytes showing the release of the first polar body were considered mature and were used for ICSI [16]. Embryos were transferred 2–5 days after oocyte retrieval.

The assessment of embryos included the cleavage rate, equality of blastomeres, the degree of fragmentation, and mononuclearity in blastomeres. Embryos were classified as follows: (1) Grade 1 embryos, defined as 4–6 cells on day 2, 8–10 cells on day 3, equal, fragmentation <20%, and no multinucleated blastomeres; (2) Grade 2 embryos, defined as 3 or >6 cells on day 2, 6–7 or >10 cells on day 3, equal or less equal, fragmentation 10–20%, and no multinucleated blastomeres; and (3) Grade 3 or 4 embryos, defined as 0–2 cells on day 2, 1–5 cells on day 3, unequal, fragmentation >20%, with or without multinucleated blastomeres. Embryo assessment was performed according to embryo quality on day 3. Grade 1 and 2 embryos were considered viable embryos, and Grade 1 embryos were defined as high-quality embryos. Finally, serum β-HCG was tested after 14 days of transfer, and an ultrasound was performed 28 days after transfer if blood β-HCG was positive.

## Statistical analysis

Multiple linear regression was used to analyze the effect of paternal age on embryo quality [i.e., numbers of fertilized oocytes, 2 pronucleus zygotes (2PNs), viable embryos, and high-quality embryos], after being adjusted for maternal age and sperm origin. Results were expressed as regression coefficient (RC) values, 95% confidence intervals (CIs), and *P* values. To evaluate the relationship between paternal age and pregnancy outcomes (clinical pregnancy, ongoing pregnancy, early pregnancy loss, and live birth), binary logistic regression was used and adjusted for maternal age, number of transferred embryos, and day of embryo transfer. Results were expressed as odds ratios (ORs), 95% CIs, and *P* values. SPSS 21.0 software was used and *P* < 0.05 was considered statistically significant.

## Results

A total of 2,627 ICSI cycles were included in this study (Table 1). Paternal age ranged from 22–78 y, with a mean of 33.57 y (SD 5.35). The group of older men included 22 men aged 50–59 y, and 5 men aged 60–78 y. Paternal age was <30 y in 589 cycles, 30–34 y in 1052 cycles, 35–39 y in 662 cycles, 40–44 y in 250 cycles, 45–49 y in 47 cycles, and ≥50 y in 27 cycles. The average maternal age was 30.46 years (SD 3.83).

**Table 1:** Cycle characteristics in the different paternal age groups.

Paternal age (years)	<30	30–34	35–39	40–44	45–49	≥50	Total
Paternal Age							
N	589	1052	662	250	47	27	2627
Mean±SD	27.42±1.60	32.03±1.39	36.69±1.38	41.42±1.24	46.43±1.38	56.33±7.77 33.57±5.35	
Maternal age							
N	589	1052	662	250	47	27	2627
Mean±SD	26.67±2.62	30.08±2.75	32.77±3.18	33.99±3.45	33.43±3.65	33.26±3.72 30.46±3.83	
Etiology							
Male factors, % (n)	70.1 (413)	62.9 (662)	56.2 (372)	54.0 (135)	44.7 (21)	59.3 (16)	61.6 (1619)
Female factors, % (n)	5.8 (34)	9.9 (104)	12.5 (83)	14.0 (35)	17.0 (8)	14.8 (4)	10.2 (268)
Both factors, % (n)	24.1 (142)	27.2 (286)	31.3 (207)	32.0 (80)	38.3 (18)	25.9 (7)	28.2 (740)
Primary infertility, % (n)	85.9 (506)	79.0 (831)	70.2 (465)	58.0 (145)	51.1 (24)	33.3 (9)	75.4 (1980)
Sperm from TESA, % (n)	8.3 (49)	6.3 (66)	7.1 (47)	9.2 (23)	6.4 (3)	14.8 (4)	7.3 (192)
Sperm from PESA, % (n)	27.0 (159)	17.0 (179)	16.2 (107)	12.8 (32)	19.1 (9)	11.1 (3)	18.6 (489)
Ejaculated sperm, % (n)	64.7 (381)	76.7 (807)	76.7 (508)	78.0 (195)	74.5 (35)	74.1 (20)	74.1 (1946)
Concentration (million/mL)							
N	139	371	251	103	19	11	894
Mean±SD	26.4±23.9	28.1±27.7	27.7±26.0	33.6±36.1	34.6±29.0	29.5±35.9	28.5±27.9
Motility (a+b)							
N	102	299	212	79	13	9	715
Mean±SD	23.2±17.4	21.9±15.7	23.5±16.6	22.2±13.7	23.1±16.7	25.4±12.1	22.7±16.0
Fertilization rate							
N	589	1052	662	250	47	27	2627
%	73.0	71.9	73.0	74.0	66.8	69.0	72.5
Embryo transfer day							
N	589	1052	662	250	47	27	2627
Mean±SD	2.96±0.50	2.92±0.46	2.90±0.53	2.87±0.57	2.94±0.44	2.96±0.59	2.92±0.50
Number of embryos transferred							
N	589	1052	662	250	47	27	2627
Mean±SD	1.94±0.35	1.98±0.38	2.05±0.49	2.13±0.55	2.02±0.53	2.00±0.55	2.00±0.43
Implantation rate							
N	589	1052	661	250	47	27	2626
%	39.37	33.96	27.38	26.13	33.68	25.93	32.56
Pregnancy rate							
N	589	1052	661	250	47	27	2626
%	56.71	49.14	43.57	42.00	40.43	40.74	48.51

The indication for ICSI was mostly male infertility (oligospermia, asthenozoospermia, teratozoospermia), accounting for 61.6% of cycles (Table 1). For sperm origin, 1,946 (74.1%) cycles used ejaculated sperm, 489 (18.6%) cycles used sperm obtained by PESA, and 192 (7.3%) cycles used sperm obtained by TESA (Table 1) Embryos were transferred on days 2–3 in 2,535 (96.5%) cycles, and on days 4–6 in 92 (3.5%) cycles. Data on semen were available for 1,159 men who used ejaculated sperm for fertilization, 255 (22.0%) of which had a sperm concentration of 0–5 sperms/high power field.

**Fig 1:**
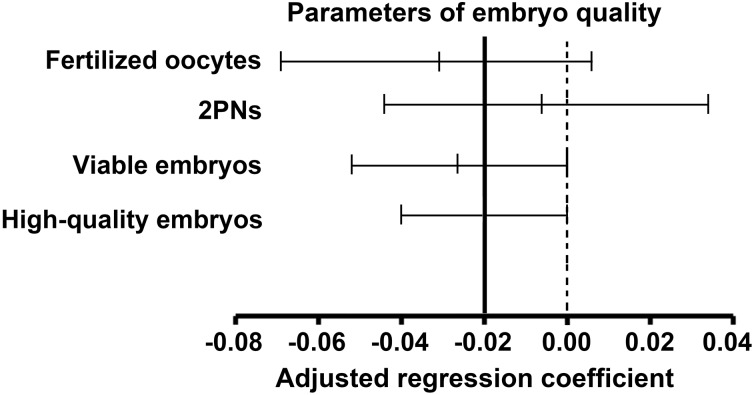
Effects of paternal age on embryo quality. Paternal age negatively affected the number of high-quality embryos (*P* < 0.05), but had no influence on other parameters of embryo quality after adjustment for maternal age and sperm origin (*P* > 0.05).

**Fig 2:**
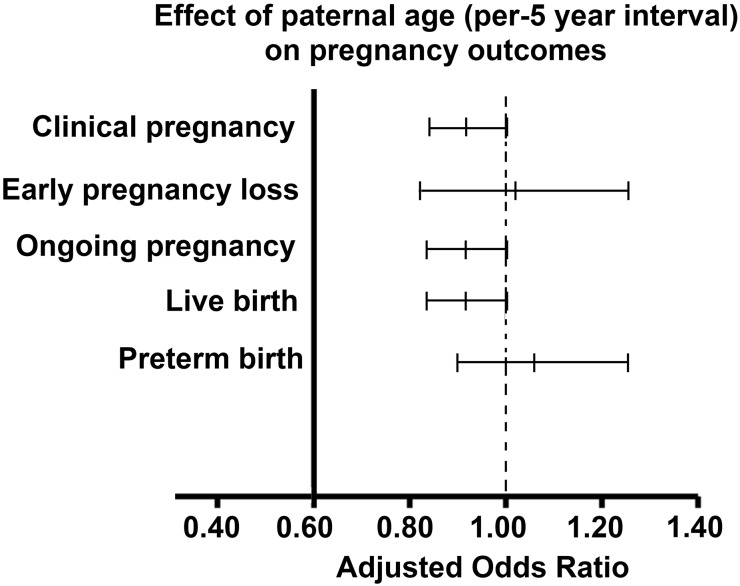
Effects of paternal age on pregnancy outcomes. Paternal age had no effect on pregnancy outcomes after adjustment for maternal age, the number of embryos transferred, and the day of embryo transfer (*P* > 0.05).

There was no association between paternal age and the following parameters of embryo quality: number of fertilized oocytes (B = -0.032; 95% CI -0.069–0.005; *P* = 0.088), number of 2PNs (B = -0.005; 95% CI -0.044–0.034; *P* = 0.806), and number of viable embryos (B = -0.025; 95% CI -0.052–0.001; P = 0.062). However, paternal age negatively influenced the number of high-quality embryos (B = -0.020; 95% CI -0.040–0.000; P = 0.045) (Fig 1). Moreover, paternal age had no effect on pregnancy outcomes (OR for a 5-year interval), including the rates of clinical pregnancy (OR 0.919; 95% CI 0.839–1.006; *P* = 0.067), ongoing pregnancy (OR 0.914; 95% CI 0.833–1.003; *P* = 0.058), early pregnancy loss (OR 1.019; 95% CI 0.823–1.263; *P* = 0.861), live births (OR 0.916; 95% CI 0.833–1.007; *P* = 0.070), and preterm births (OR 1.061; 95% CI 0.898–1.254; *P* = 0.485) (Fig 2). Among the 1,249 pregnancies, 10 birth defects were observed, including one in the group of paternal age <30 y, five in the group aged 30–34 y, and four in the group aged 35–39 y (Table 2). There were no statistical differences in the rates of birth defects between the three age groups (*P* > 0.05).

**Table 2:** Birth defects between different paternal age groups.

Paternal age (years)	<30	30–34	35–39	40–44	45–49	≥50	Total
Pregnancy cycles	331	510	284	105	19	11	1249
Birth defects (n)	1	5	4	0	0	0	10
Birth defects (%)	0.30^a^	0.98^a^	1.41^a^	0.00	0.00	0.00	0.80

a. There were no statistical differences in rates of birth defects between the three paternal age groups.

## Discussion

Our study found that paternal age had no impact on the number of fertilized oocytes and viable embryos, or on pregnancy outcomes using ICSI; however, increased paternal age negatively influenced the number of high-quality embryos after adjustment for female age.

Prior to this study, only six other studies focused on the influence of paternal age on embryo quality in ICSI, with contradicting findings [6–9, 19, 20]. Four [6–9] of these six previous reports illustrated that paternal age had no impact on embryo quality. Aboulgar et al. investigated the outcomes of ICSI in two age groups of men (≥50 y and <50 y) in a retrospective case-control study matched for female age, and found no significant difference in the number of grade 1 embryos; however, the fertilization rate in the younger group was significantly higher [10]. In addition, Ferreira et al. found no effect of paternal age on the rate of high-quality embryos in either an oligozoospermic group (*P* = 0.442, RC = 0.1822) or a normozoospermic group (*P* = 0.368, RC = 0.1725) [11]. Similarly, Tsai et al. reported that the parameters of embryo quality (i.e., cleavage rate, Day-2 good embryos) were not statistically different across all paternal age groups, after adjustment for female age [12]. Moreover, in a study of nearly 5,000 oocyte-donated ICSI cycles, Begueria et al. found that paternal age was not associated with the morphological embryo score, after being adjusted for semen origin (i.e., fresh versus frozen) and donor age [13]. On the other hand, Luna et al. showed that paternal age did have a detrimental effect on the number of embryos with ≥7 cells on day 3, and the blastocyst formation rate [17]. Moreover, Frattarelli et al. showed male age had no correlation with early embryo development through the cleavage stage, but negatively affected blastocyst formation [18]. In agreement with the studies by Luna et al. and Frattarelli et al., our study also showed that paternal age had a detrimental effect on the number of high-quality embryos at the cleavage stage.

When considering the influence of paternal age on pregnancy outcomes, the results of our study differ from previous studies. For example, Ferreira et al. concluded that paternal age affected pregnancy rate only in men with oligozoospermia, with a 5% of decrease in the pregnancy rate as paternal age increased by one year. They suggested that the negative influence of paternal age in oligozoospermic men was due to decreased sperm quality and genetic alterations. In particular, in oligozoospermic men with compromised spermatogenesis, important sperm-related factors (e.g., centrosomic factors and oocyte activation) may be affected by age [11]. Moreover, in a binary logistical analysis of nearly 5,000 oocyte-donated ICSI cycles, Begueria et al. demonstrated that paternal age was not related to reproductive outcomes (OR for a 5-year interval), including biochemical pregnancy (1.0; 95% CI 0.96–1.05; *P* = 0.91), miscarriage (1.06; 95% CI 0.94–1.03; *P* = 0.52), ongoing pregnancy (0.98; 95% CI 0.94–1.033; *P* = 0.52), and live births (0.98; 95% CI 0.94–1.03; *P* = 0.52) [13]. Similarly, Aboulgar et al. found no significant difference in the pregnancy rate between groups of paternal age ≥50 y and paternal age <50 y (37.9% versus 36.6%; OR = 1.06, 95% CI = 0.72−1.55) [6]. Finally, when only investigating TESE-ICSI cycles, Tsai et al. also reported no association between paternal age and any of the reproductive outcomes. They hypothesized that frozen-thawed TESE procedures could overcome the poor-quality spermatozoa related to advanced male age [12]. However, we should be precautious of this conclusion because it is difficult to reach statistical difference with limited sample size of 212 cycles.

While there are several researches about the effect of paternal age on preterm birth [19–21], data remains limited for ICSI. Zhu et al. demonstrated that older fathers had an increased risk of preterm birth (i.e., age 25–29 y, OR = 1.3; age 35–39 y, OR = 1.4; age 40–44, OR = 1.7; age 45–49 y, OR = 1.6; and age 50+, OR = 2.1) compared with those aged 20–24 y [19]. In a large study using a national data set of 1,510,823 Italian first born singletons, Astolfi et al. also found that paternal age increased the risk of preterm births: fathers aged 45–49 y showed high ORs for very preterm births of 1.91 (95% CI 1.08–3.38) and 1.72 (95% CI 1.25–2.36) in mothers aged 20–24 y and mothers aged 25–29 y, respectively[20]. Conversely, our research showed no relationship between paternal age and preterm birth rates, which may be explained by the limited sample size. Therefore, further study on the influence of paternal age on preterm birth in ICSI cycles with a national database is required.

Unlike paternal age, many studies have investigated the influence of maternal age on reproductive outcomes of ART. Advanced maternal age increases the likelihood of aneuploidy of oocytes and miscarriages, and decreases the pregnancy and live birth rates. Based on the fact that maternal age affects ART outcomes, a major limitation of this type of study becomes the difficulty in controlling for the maternal age. To limit this effect, only cycles in which maternal age was <39 y and embryos were available for transfer were included in this study. Moreover, when we analyzed the effect of paternal age on embryo quality and pregnancy outcomes, maternal age was included as a variable to control the bias from maternal age.

In 61.6% of couples undergoing ICSI in this study, the etiology was male infertility. This is not surprising as ICSI is not our first choice for fertilization, and is only used in our clinic when there are certain indications (such as severe oligospermia, asthenozoospermia, and teratozoospermia) or a previous failure in IVF. Indeed, the percentage of couples undergoing ICSI in our center is typically only 20%, compared to 80% undergoing IVF cycles. In addition, cytogenetic analysis of semen specimens indicated that the frequency of numerical and structural aberrations was significantly greater in chromosomes of older donors aged 59–74 y compared with the control group (aged 23–39 y) [22]. Despite this, our study, as well as most previous studies, illustrated limited influence of paternal age on reproductive outcomes of ICSI cycles. However, the sample size of older men (>60 y) was extremely limited in our study. Indeed, while Begueria et al. included 69 men aged >60 y in their study [13], Aboulghar et al. only included 29 [10], and our study only had five. Moreover, Ferreira et al. [11] did not mention any details about the paternal age, and in the study by Tsai et al., the upper limit was only 51 y [12]. Therefore, the differing numbers of older men aged >60 y included in the various studies may explain the observed discrepancies of the results.

Finally, while previous studies confirmed that paternal age had an impact on psychiatric and birth defects, unfortunately we were not able to make these conclusions in our study, as the follow-up was limited to the time of birth. Therefore, more studies are necessary on the long-term effect of paternal age on offspring heath.

In conclusion, increased paternal age negatively influenced the number of high-quality embryos after being adjusted for female age but had no effect on pregnancy outcomes in couples undergoing ICSI cycles. In future, more studies, that include older men aged over 60 years and a longer-term follow-up, are needed.
